# The Management of Osteoporosis in Chronic Kidney Disease: A Review of Diagnostic and Therapeutic Approaches

**DOI:** 10.7759/cureus.73882

**Published:** 2024-11-17

**Authors:** Fatima Tariq, Mehjabeen Ahmad, Muhammad Subhan, Syed Muhammad Zaid Alvi, Muhammad Umar Tariq, Sami Ullah, Asma Khalid, Ruqiya Bibi, Muaz Shafique Ur Rehman, Ayesha Abbas

**Affiliations:** 1 Internal Medicine, University of Health Sciences, Lahore, PAK; 2 Internal Medicine, Nishtar Medical University and Hospital, Multan, PAK; 3 Medicine, Allama Iqbal Medical College, Lahore, PAK; 4 Medicine, Ziauddin Medical College, Karachi, PAK; 5 Medicine, King Edward Medical University, Lahore, PAK; 6 Internal Medicine, King Edward Medical University, Lahore, PAK; 7 Internal Medicine, Jinnah Hospital, Lahore, Lahore, PAK; 8 Medicine, Akhtar Saeed Medical and Dental College, Lahore, PAK

**Keywords:** bisphosphonates, bone mass, chronic kidney disease (ckd), ckd-mineral and bone disorder (ckd-mbd), denosumab, fracture risk, odanacatib, osteoporosis, romosozumab, teriparatide

## Abstract

Chronic kidney disease (CKD) has shown a growing association with osteoporosis, comprising part of the broader CKD-mineral and bone disorder (CKD-MBD). CKD-MBD is marked by alterations in calcium, phosphorus, parathyroid hormone (PTH), and vitamin D metabolism, significantly elevating fracture risk. While traditional osteoporosis treatments such as bisphosphonates, denosumab, and teriparatide have been adapted for CKD patients, recent innovations have introduced agents aimed at enhancing bone mass and reducing fracture incidence. This study aims to evaluate the pathophysiology, diagnostic methods, and tailored management strategies for osteoporosis in CKD patients. A detailed review of the literature was conducted, involving an in-depth search of PubMed, Medical Literature Analysis and Retrieval System Online (MEDLINE), and the Cochrane Library databases for studies published between 2017 and 2024. Studies were selected based on inclusion criteria focusing on CKD-related osteoporosis, diagnostic criteria, and treatment outcomes. Data extraction and quality assessment were independently performed by multiple reviewers to ensure thoroughness and reduce bias. Findings highlight that conventional treatments, such as bisphosphonates, denosumab, and teriparatide, when tailored to CKD stages, demonstrate variable effectiveness in lowering fracture risk.

Additionally, emerging pharmacologic agents hold promise in improving bone density, though evidence on these newer therapies remains limited. Osteoporosis management in CKD patients necessitates a personalized approach guided by the disease's stage and individual profile. This review underscores the potential of emerging therapies and emphasizes the need for further research to refine treatment protocols, aiming to enhance patient outcomes in this complex population.

## Introduction and background

Chronic kidney disease (CKD) is a widespread condition characterized by gradual reductions in kidney function [1,2]. Measurements that fall below 60 mL/minute/1.73 m² over at least three months typically indicate kidney disease [1]. The stages of CKD can be broken down according to glomerular filtration rate (GFR) levels, from stage 1 (GFR ≥ 90) to stage 5 (kidney failure, GFR ≤ 15) [2]. CKD is a progressive condition that significantly impacts bone metabolism, leading to a complex spectrum of skeletal disorders collectively known as CKD-mineral and bone disorder (CKD-MBD) [3,4]. In advanced stages of CKD (stages 3a-5D), renal dysfunction disrupts critical metabolic pathways, including calcium and phosphorus homeostasis, parathyroid hormone (PTH) regulation, and vitamin D metabolism [4]. These disturbances compromise bone remodeling processes, resulting in reduced bone mass, structural deterioration, and increased fracture risk, particularly in sites such as the hip, where fractures are associated with heightened morbidity and mortality [4]. CKD-MBD encompasses disorders in calcium, phosphorus, parathyroid hormone (PTH), vitamin D metabolism, and bone turnover dynamics [5].

Pathophysiologically, CKD-related osteoporosis involves elevated PTH levels, the retention of phosphate, irregular calcium handling, vitamin D deficiency, soft tissue calcification, and decreased bone formation and turnover [6]. Osteoporosis in CKD includes both high-turnover bone disease (secondary hyperparathyroidism) and low-turnover bone disease (adynamic bone disease), with prevalence increasing as CKD severity progresses [5,6]. High-turnover bone disease is characterized by excessive bone resorption due to elevated PTH levels, often resulting from phosphate retention and vitamin D deficiency [6]. In contrast, low-turnover bone disease involves reduced bone formation and mineralization, leading to adynamic bone disease [5,6]. These disturbances in bone turnover dynamics are directly linked to the metabolic imbalances caused by renal dysfunction [7]. Osteoporosis in CKD disproportionately affects postmenopausal women due to the decline in estrogen levels, which plays a pivotal role in maintaining bone density [6]. Estrogen deficiency exacerbates CKD-related bone loss by enhancing osteoclastic activity, reducing osteoblastic function, and increasing parathyroid hormone levels [6,7]. Furthermore, hormonal fluctuations in CKD patients, particularly decreased estrogen, contribute to the development of CKD-MBD [8].

Hormonal therapies, such as estrogen replacement therapy, may be considered in CKD management; however, their interaction with CKD-MBD treatment requires careful evaluation, as estrogen may impact vitamin D and phosphate metabolism and influence the efficacy of calcimimetics and other osteoporosis medications [8]. Clinical manifestations can include bone pain, fractures (hip and vertebral fractures in particular), reduced mobility, and overall quality of life issues that significantly impact quality of life [9]. Current statistical information indicates that up to 27% of CKD patients with osteoporosis and stage 3 CKD are estimated to have GFR below 35 mL/minute/1.73 m² [10]. Central dual-energy X-ray absorptiometry (DEXA) remains the gold standard in measuring bone mineral density (BMD) [9,10]. Management strategies for osteoporosis in CKD aim to address bone health concerns and potential MBD complications related to their disease progression [10,11]. Bisphosphonates are frequently prescribed, yet care must be taken when administering them to patients with creatinine clearances (CrCl) above 35 mL/minute due to potential side effects [12]. Alternative treatments such as denosumab or teriparatide should be considered if clearance levels drop, considering individual patient profiles and risk factors [13]. This study is designed to comprehensively investigate the pathophysiology, diagnostic approaches, therapeutic interventions, and preventive strategies of osteoporosis in CKD patients by evaluating prevalence trends, identifying risk factors, assessing treatment efficacy, and creating management guidelines [14].

## Review

Methodology

Search Strategy

This review employed a systematic literature search to collect relevant studies on the management of osteoporosis in chronic kidney disease (CKD) patients, covering publications from January 2016 to October 2024. The databases searched included PubMed, Medical Literature Analysis and Retrieval System Online (MEDLINE), Embase, and the Cochrane Library. Specific search terms and Boolean operators were used to ensure the comprehensive retrieval of relevant studies. Terms included "osteoporosis", "chronic kidney disease", "CKD", "bone mineral density", "bisphosphonates", "denosumab", "romosozumab", "abaloparatide", "teriparatide", "sclerostin inhibitors", "cathepsin K inhibitors", and "fracture risk". Boolean operators (AND and OR) were systematically applied, and additional database filters were set to include only English-language studies published within the specified date range. This approach was intended to maximize the precision of the search and improve replicability for future researchers.

Inclusion and Exclusion Criteria

Studies were included if they specifically addressed osteoporosis management in CKD patients and met the following criteria: published between January 2016 and October 2024; designed as clinical trials, observational studies, meta-analyses, or systematic reviews; reported on relevant outcomes such as changes in BMD, fracture risk, or safety profiles associated with osteoporosis treatments; and included data relevant to CKD stages to assess treatment outcomes in different disease stages. The exclusion criteria applied were non-English language publications, studies that focused on non-CKD populations, and articles without pertinent outcomes, such as editorials, opinion pieces, or case reports.

Data Extraction and Quality Assessment

Data extraction was performed using a standardized form to ensure consistency across studies. Information collected included study design, patient demographics, sample size, intervention specifics, outcomes measured, and key findings. Each study underwent quality assessment by two independent reviewers to enhance the robustness and minimize bias in the evaluation process.

For quality assessment, the Cochrane Risk of Bias tool (The Cochrane Collaboration, London, UK) was applied to randomized controlled trials, assessing domains such as randomization, blinding, and completeness of outcome data. The Newcastle-Ottawa Scale was used for observational studies, with a focus on selection, comparability, and outcome assessment criteria. Individual bias scores or summaries were documented to increase transparency and ensure that only high-quality studies were included in the final synthesis.

Data Synthesis

**Figure 1:**
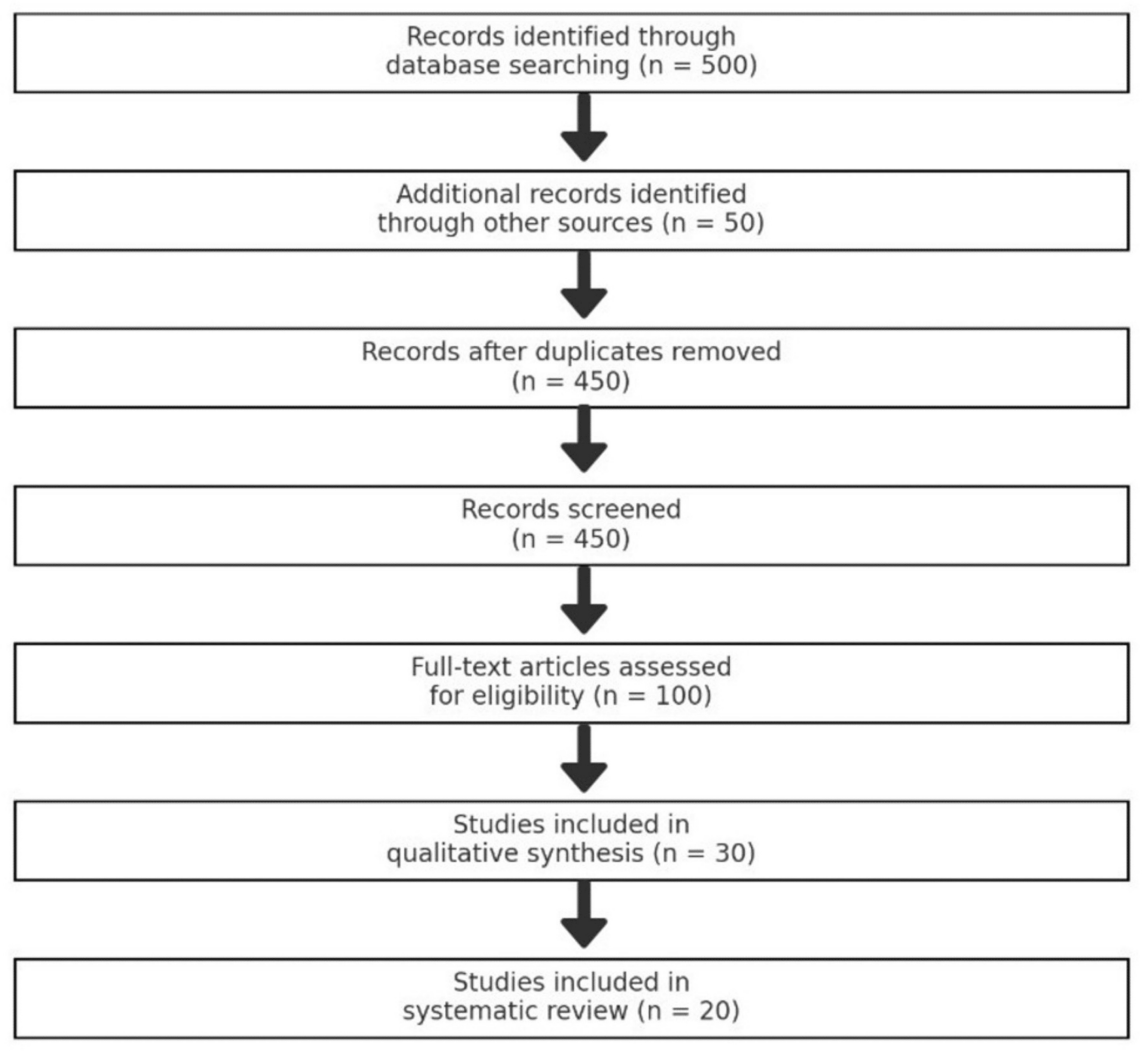
PRISMA Flowsheet of Selected Studies PRISMA: Preferred Reporting Items for Systematic Reviews and Meta-Analyses

The data were synthesized qualitatively and categorized by intervention type, such as antiresorptive medications, anabolic agents, and emerging therapies. Treatment effects were also stratified by the CKD stage to provide insights into the outcomes for each disease stage. Although this narrative review provides a descriptive synthesis of the literature, we included a Preferred Reporting Items for Systematic Reviews and Meta-Analyses (PRISMA) flow diagram to illustrate the study selection process and highlight the scope of the literature analyzed. Figure 1 depicts the PRISMA flowsheet of selected studies.

Discussion

As part of managing osteoporosis in patients with CKD, it is crucial to consider all its stages, which significantly influence bone metabolism [1-4]. The interdependency between CKD progression and bone health presents unique difficulties when diagnosing and treating osteoporosis [3,4]. As CKD progresses, mineral metabolism and hormone production changes further complicate bone density assessments and fracture risk predictions [3,4]. This section will discuss these challenges, with particular emphasis on diagnosing unique considerations for those living with CKD, managing osteoporosis specifically among this population, and antiresorptive drugs' ability to combat bone loss while considering individual needs and risks associated with CKD. Table 1 highlights the various stages of CKD.

**Table 1:** CKD Stages Based on GFR and Albuminuria CKD, chronic kidney disease; GFR, glomerular filtration rate

CKD Stage	GFR (mL/minute/1.73 m²)	Albuminuria Category
1 (normal)	≥90	A1 (normal: <30 mg/g)
2 (mild decline)	60-89	A1 (normal: <30 mg/g) or A2 (mild: 30-300 mg/g)
3A (mild-to-moderate decline)	45-59	A1 (normal: <30 mg/g), A2 (mild: 30-300 mg/g), or A3 (moderate to severe: >300 mg/g)
3B (moderate-to-severe decline)	30-44	A2 (mild: 30-300 mg/g) or A3 (moderate to severe: >300 mg/g)
4 (severe decline)	15-29	A2 (mild: 30-300 mg/g) or A3 (moderate to severe: >300 mg/g)
5 (kidney failure)	≤15	A3 (moderate to severe: >300 mg/g)

Navigating the Intricacies of Bone Metabolism in Chronic Kidney Disease

Understanding and treating CKD-MBD requires an exploration of bone mineral metabolism, specifically how the complex interactions between calcium and phosphate regulation manifest themselves [11]. CKD alters bone metabolism through increased PTH levels, calcium, vitamin D, phosphate, and fibroblast growth factor-23 (FGF-23)/Klotho dynamics, decreasing bone strength and increasing fracture susceptibility [11]. Klotho, a crucial membrane protein in renal proximal and distal tubules, maintains phosphate and vitamin D homeostasis, and its decreased expression in early CKD stages correlates with elevated FGF-23 levels, leading to increased urinary phosphate excretion and diminished calcitriol production [10]. Klotho deficiency exacerbates CKD-MBD by impairing phosphate regulation, disrupting vitamin D signalling, and promoting secondary hyperparathyroidism, which initially compensates for diminished calcitriol but eventually fails in CKD stages 4-5, resulting in uncontrolled hyperphosphatemia, severe secondary hyperparathyroidism, calcitriol deficiency, accelerated vascular calcification, and increased morbidity and mortality [10,11]. Restoring Klotho expression or mimicking its functions may offer therapeutic potential for managing CKD-MBD, addressing complications such as osteitis fibrosa cystica, vascular calcification, and cardiovascular risk [12,13].

**Figure 2:**
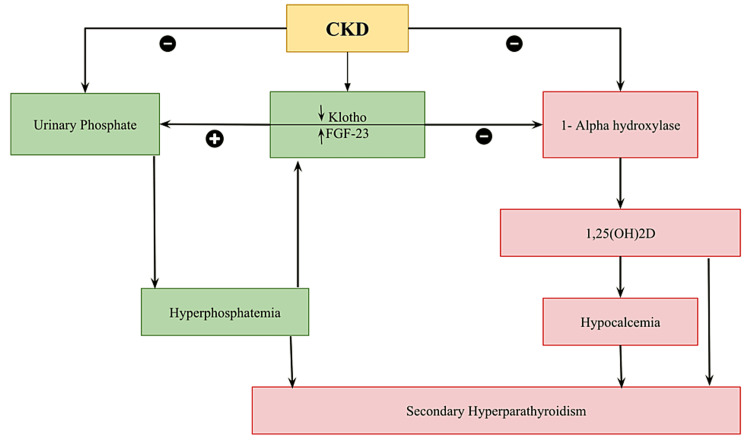
Pathogenesis of Chronic Kidney Disease (CKD) 1,25(OH)2D, 1 alpha, 25-dihydroxyvitamin D3; FGF-23, fibroblast growth factor-23

**Figure 3:**
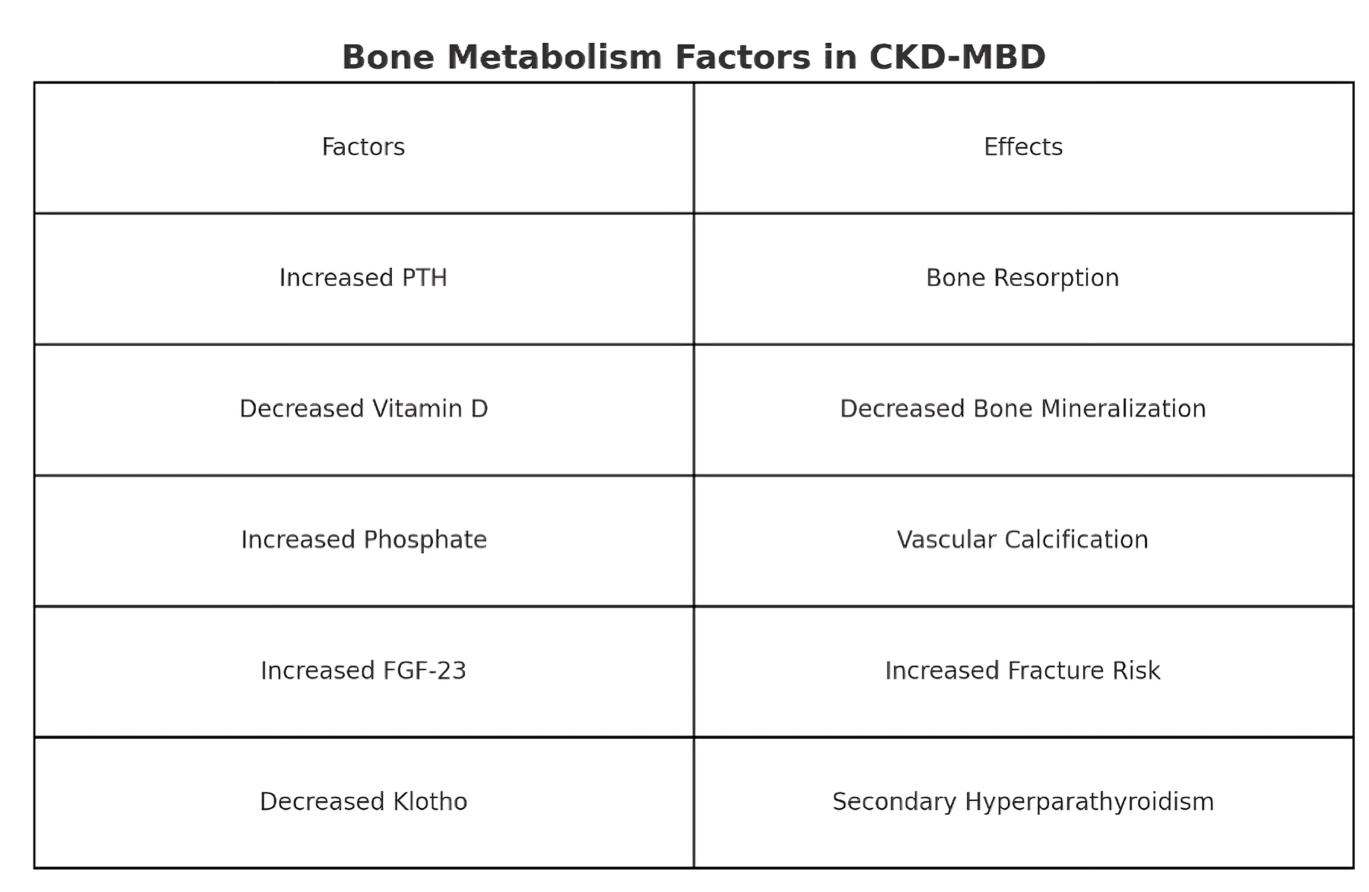
Bone Metabolism Factors in CKD-MBD CKD-MBD, chronic kidney disease-mineral bone disease; PTH, parathyroid hormone; FGF-23, fibroblast growth factor-23

FGF-23 is an essential mediator, initiating phosphate retention in response to declining renal function. Independent of PTH, FGF-23 enhances renal phosphate excretion while suppressing 1-alpha hydroxylase production, exacerbating calcitriol deficiency [13,14]. As GFR drops below 20-30 mL/minute, compensatory mechanisms become ineffective, leading to hyperphosphatemia [14]. Hyperphosphatemia, deficient calcitriol levels, and hypocalcemia increase PTH production and secretion [14,15]. Elevated PTH levels seek to normalize blood phosphate levels by increasing renal phosphate excretion and correcting hypocalcemia through 1-alpha hydroxylase activation [15]. PTH's primary role is restoring normal bone turnover by treating hypocalcemia [15]. PTH is integral to bone turnover by acting alongside phosphate, calcitriol, systemic factors, FGF-23, and growth hormones [15]. Phosphorus can inhibit osteoclast activity regardless of PTH levels, while FGF-23 inhibits osteoblast maturation and mineralization, further complicating bone metabolism [16,17]. Proper bone remodeling relies on optimal levels of calcitriol; deficiencies of this hormone have been associated with reduced bone metabolism [15-17]. Fluctuations in serum calcium levels, whether hypo- or hypercalcemia, also impact bone turnover dynamics [18]. Progressing renal disease exposes individuals to proinflammatory cytokines such as tumor necrosis factor (TNF), interleukin-1 (IL-1), and IL-6, exacerbating bone turnover abnormalities [18]. This intricate interaction compounds the complexity of CKD-MBD, underscoring its need for comprehensive management strategies focused on calcium-phosphate balance and bone health [12-18]. Figure 2 depicts the pathogenesis of CKD, while Figure 3 shows the key factors in CKD-BMD and their effects on it.

Challenges and Considerations in Diagnosing and Managing Osteoporosis in Chronic Kidney Disease

Diagnosing osteoporosis in CKD presents unique challenges [1-6]. DEXA scans, recommended by the World Health Organization (WHO), provide one assessment method [19]. This method measures BMD at sites such as the lumbar spine or femoral neck, classifying patients into various treatment groups based on fracture risk as measured by T-score classification [19]. Importantly, this classification system has only been validated in White women; its applicability to men, children, ethnic groups, and CKD populations remains to be assessed [19]. The National Institutes of Health (NIH) takes an alternative view and explains osteoporosis as a condition marked by decreased bone strength leading to increased fracture risk [20].

Although widely utilized, the WHO criteria for osteoporosis diagnosis focus heavily on BMD measurements that may not fully represent bone quality or architecture [20]. This limitation is especially prominent among patients living with CKD, where bone quality rather than density alone may better predict fracture risk [21]. DEXA imaging may be insufficient for diagnosing CKD-MBD due to the intricate alterations in bone metabolism seen in CKD, which can lead to misinterpretations and less effective therapeutic choices [22-24]. While BMD measurements may have some correlation with clinical outcomes in CKD patients, BMD alone has not proven to be a reliable predictor of fracture risk in this population, especially as the severity of kidney disease progresses [22-24]. In advanced CKD stages, BMD assessments become less dependable, indicating the need for more sophisticated diagnostic modalities such as bone biopsy with quantitative histomorphometry and quantitative computed tomography (QCT) [25].

Bone biopsies, considered the gold standard for diagnosing and monitoring renal osteodystrophy in CKD patients, are rarely performed in clinical practice due to associated risks and challenges [22,23]. Studies show that bone biopsies are utilized in less than 5% of CKD patients [21,22]. The procedure carries risks of infection (2.5%-5%), bleeding (2%-5%), and nonhealing due to poor bone turnover [23,24]. Additionally, CKD patients' compromised bone quality and altered mineral metabolism increase the risk of biopsy-related complications [24,25]. A systematic review of 22 studies found that only 2.6% of CKD patients underwent bone biopsy, highlighting the need for alternative diagnostic methods [25,26]. Despite these limitations, bone biopsies remain essential for accurate diagnosis and treatment monitoring in select cases [21]. Bone biopsies are essential for providing detailed insights into bone architecture, microarchitecture, osteoid composition, and mineralization patterns, offering a valuable guide for targeted treatment strategies [19]. Additionally, while biochemical markers are commonly used to evaluate bone turnover, their utility in CKD patients has shown limited effectiveness [19]. To enhance diagnostic precision and improve outcomes in CKD-MBD management, there is a clear need to integrate advanced imaging methods such as QCT and routinely consider bone biopsies alongside traditional techniques [20].

The US National Health and Nutrition Examination Survey (NHANES) revealed that 27% of osteoporosis patients had stage 3 CKD, with an estimated GFR below 35 mL/minute/1.73 m² [21,22]. The management of osteoporosis among those living with chronic kidney disease (CKD), particularly if they also suffer from mineral and bone disorder (MBD), requires a carefully balanced approach that considers both the unique pathophysiology of bone loss in CKD-MBD and the potential impact of traditional osteoporosis treatments on renal function and calcium-phosphate metabolism [21,22]. CKD-MBD includes abnormalities in calcium, phosphorus, PTH, and vitamin D levels; bone turnover, mineralization, and volume growth strength; and vascular or soft tissue calcification [21,22]. Since bone disorders are frequently linked with vasculature calcifications, treatments to improve bone health may also affect vasculature [21,22]. Lifestyle interventions for all those at high risk for fractures, including those living with CKD, are crucial [21,22]. These parameters include adequate calcium and vitamin D intake, regular exercise, quitting smoking, limiting alcohol consumption, and fall prevention [21,22]. We briefly explore these strategies below within the context of CKD but more comprehensively elsewhere [21,22].

An optimal diet to prevent fractures should include sufficient calories, calcium, and vitamin D [20-22]. Individuals with an estimated glomerular filtration rate (eGFR) of ≥30 mL/minute/1.73 m² who do not exhibit biochemical evidence of CKD-MBD should consume calcium and vitamin D similarly to those without CKD [20-22]. Additional calcium and vitamin D supplementation recommendations can be found elsewhere [19-21]. However, for patients with an eGFR of 30 mL/minute/1.73 m², an adequate total calcium intake should reach 1,200 mg daily with no more than 500 mg from supplements [19-21]. Dietary calcium sources such as calcium-fortified orange juice, soy products, and vegetables may provide enough calcium [20,21]. Due to dairy's high phosphorus content, nondairy sources may be preferred over dairy, and daily supplementation of 800 IU of vitamin D (cholecalciferol or ergocalciferol) is advised [20,21]. Unfortunately, however, its impact on fracture risk or falls among individuals with CKD who have an eGFR of less than 30 mL/minute/1.73 m² remains poorly understood [22-24]. These recommendations are based on low-quality evidence and extrapolated from patients without severe CKD [22].

Studies in postmenopausal women suggest that supplementing vitamin D could reduce falls and hip fractures, suggesting potential benefits for those living with severe CKD [23]. Excess calcium supplementation in patients with an eGFR of less than 30 mL/minute/1.73 m² may increase their risk of arterial calcification and cardiovascular disease [24]. This risk is linked with hyperphosphatemia, hyperparathyroidism, and elevated fibroblast growth factor-23 (FGF-23) [24]. Hypercalcemia from vitamin D supplementation typically manifests itself when serum 25-hydroxyvitamin D levels exceed 150 ng/mL [25]. However, this adaptation is impaired in patients with eGFR of <60 mL/minute/1.73 m², and lower serum 25-hydroxyvitamin D levels may cause hypercalcemia [26]. Therefore, the close monitoring of serum calcium is essential, especially when eGFR is <30 mL/minute/1.73 m² [26].

Fall prevention is essential for patients with an eGFR of less than 15 mL/minute/1.73 m², who tend to be frail and vulnerable to falling [27]. Furthermore, severe CKD patients often exhibit signs of sarcopenia, a condition characterized by muscle mass deficiency and reduced strength [28]. Reducing muscle tone, strength, and balance deficits is a key non-pharmacologic strategy for managing osteoporosis in CKD patients [28]. Simple office-based tests such as handgrip strength or gait speed screenings can detect muscle weakness [28]. Patients unable to rise from a chair without using their hands are at increased risk of falls [27,28]. Intervention strategies include targeted physical therapy and core-strengthening exercises [28]. Osteoporosis treatment should focus on preventing fractures [28]. Solely based on fracture risk assessment based on the history of fragility fractures, BMD measurements, and the presence/absence of CKD-MBD [28,29], pharmacologic therapy can effectively select patients for therapy [28,29]. Before considering pharmaceutical therapy to address fractures or low BMD, CKD-MBD must first be eliminated as a possible cause through biochemical testing or bone biopsy [28,29].

Our approach aligns with the guidelines outlined by the 2017 Kidney Disease: Improving Global Outcomes (KDIGO) [30]. Individuals with GFR greater or equal to 30 mL/minute/1.73 m² who do not demonstrate evidence of CKD-MBD can use the same criteria for selecting pharmaceutical therapies [30]. Individuals with low BMD, GFR of less than 30 mL/minute/1.73 m² (or an eGFR of ≥30 mL/minute/1.73 m²), no history of fragility fractures, and GFR of less than 30 mL/minute/1.73 m² should not receive osteoporosis therapy [30,31]. It is essential to manage and monitor secondary hyperparathyroidism and abnormalities in mineral metabolism [30,31]. The decision to perform a bone biopsy to rule out renal osteodystrophy before initiating osteoporosis therapy is left to the discretion of the specialist in metabolic bone diseases [30,31]. Before initiating osteoporosis therapy, performing a bone biopsy to rule out renal osteodystrophy should be left up to the discretion of a specialist in metabolic bone diseases [30,31]. Pharmacologic therapy may be considered in individuals whose GFR falls between 15 and 30 mL/minute/1.73 m² with fragility fractures but no evidence of renal osteodystrophy [30-32]. Individuals with GFR of less than or equal to 15 mL/minute/1.73 m² who present with fragility fractures but no evidence of renal osteodystrophy may benefit from pharmacologic therapy when their risk for mortality from repeated fractures (e.g., hip fractures) is high [30-32]. Patients with an eGFR of 15 mL/minute/1.73 m² have an increased incidence of CKD-BMD, so conducting a bone biopsy to evaluate for renal osteodystrophy should be considered before considering antiresorptive therapy [32]. If a bone biopsy is not possible, elevated bone-specific alkaline phosphatase (BSAP) levels and intact serum PTH levels of over 350 pg/mL suggest an absence of dynamic bone disease and that treatment with an antiresorptive osteoporosis agent might be effective [32,33].

Management of Osteoporosis in CKD

Pharmacotherapy is the cornerstone of osteoporosis treatment in general population settings, using medications such as bisphosphonates and anabolic agents such as estrogen receptor modulators and bone formation stimulators such as teriparatide to manage primary osteoporosis [10]. Osteoporosis guidelines also recommend active vitamin D and calcium supplementation, although due to some medications being cleared out via the kidneys, this may pose challenges in treating CKD patients [10].

Antiresorptive Medications

Bisphosphonates such as alendronate and risedronate have become standard therapies for osteoporosis treatment; however, they come with increased risks in people living with CKD due to renal excretion [10]. One study revealed an accumulation of bisphosphonates in dialysis patients treated with these drugs, raising concerns of "frozen bone," possibly due to inadequate bone turnover or reduced variability in material properties [11-15]. Recent research, such as a European Calcified Tissue Society review, highlights the complexity and risks associated with bisphosphonate use for managing bone health in CKD patients [11-15]. Bisphosphonates, commonly used to treat osteoporosis and renal osteodystrophy, can paradoxically contribute to "frozen bone" in CKD patients with reduced bone turnover [11,12]. This phenomenon is attributed to bisphosphonates' potent inhibition of osteoclast-mediated bone resorption, leading to an imbalance in bone remodeling [12]. Bisphosphonates can further suppress bone formation in CKD patients with pre-existing low bone turnover, resulting in adynamic bone disease [12,13].

Studies demonstrate that bisphosphonates decrease osteoid surface and thickness, reducing bone formation rates [13,14]. Additionally, bisphosphonates' long half-life and persistence in bone tissue can prolong their suppressive effects, exacerbating reduced bone turnover [14]. A systematic review of 15 studies found that CKD patients treated with bisphosphonates significantly increased adynamic bone disease prevalence [15]. Clinicians should exercise caution when prescribing bisphosphonates to CKD patients, particularly those with reduced bone turnover, and closely monitor bone biopsy results to avoid "frozen bone." Recent meta-analyses conducted in 2022 explored the efficacy of bisphosphonates (including alendronate) for treating CKD patients [16]. The study determined that alendronate increased BMD at the hip, femoral neck, and spine while raising serum creatinine levels slightly, suggesting potential renal issues [16]. A 2017 study on risedronate also demonstrated significant increases in BMD and reductions in fracture rates across various levels of renal impairment without an increase in adverse renal events [17]. However, these studies often excluded patients with severe CKD stages, leaving us without an accurate picture of bisphosphonates' efficacy in preventing fractures among more advanced stages [16-18]. Risedronate was discovered to significantly increase BMD and reduce fracture rates across different levels of renal impairment without considerably increasing adverse renal events [17].

Denosumab

Denosumab, a monoclonal antibody targeting the nuclear factor-kappa B receptor activator, offers an alternative as the kidneys do not clear it, making it suitable for individuals with creatinine clearance below 35 mL per minute [19]. It has been shown to enhance BMD and prevent fractures, with its effectiveness reportedly unaffected by renal function [20]. Recent studies continue to underscore the efficacy and safety of denosumab in managing osteoporosis, even in patients with CKD [21-25]. This population is particularly vulnerable due to impaired renal calcium reabsorption and prevalent vitamin D deficiency [21,22]. Studies demonstrate that denosumab increases the risk of hypocalcemia in CKD patients, with incidence rates ranging from 10% to 30% [22,23]. A meta-analysis of 12 trials found that CKD patients treated with denosumab had a 3.5-fold increased risk of hypocalcemia compared to those with normal renal function [24]. Moreover, severe hypocalcemia (<8 mg/dL) has been reported in up to 5% of CKD patients receiving denosumab [25]. Clinicians should closely monitor serum calcium levels and consider calcium and vitamin D supplementation to mitigate this risk in CKD patients treated with denosumab [25,26]. Miyaoka et al.'s (2019) study included osteoporotic patients with normal kidney function and aimed to evaluate the effects of denosumab on GFR and serum phosphorus levels [23]. The study found that denosumab treatment significantly improved GFR and lowered serum phosphorus levels, both beneficial for kidney function [23].

Additionally, denosumab was effective in increasing BMD at various skeletal sites, further supporting its efficacy in treating osteoporosis [23]. The study concluded that denosumab enhances bone health and positively affects kidney function by reducing serum phosphorus levels in individuals with normal kidney function [23]. In a meta-analysis published in 2023, denosumab demonstrated substantial reductions in nonvertebral fractures by 20%, hip fractures by 40%, and vertebral fractures by 68% in individuals with a GFR as low as 30 mL/minute/1.73 m² [26]. However, despite these benefits, denosumab can induce hypocalcemia, particularly in CKD patients, necessitating careful monitoring and the use of active vitamin D to maintain calcium balance [26].

Raloxifene

Raloxifene, a selective estrogen receptor modulator, is another antiresorptive agent that has shown efficacy in increasing BMD in postmenopausal women with CKD [27]. Studies have linked higher BMD with reduced creatinine clearance [28-30]. Additionally, raloxifene has been associated with a slower progression of renal diseases and fewer kidney-related adverse events [31]. However, it does come with a risk of decreased serum calcium and increased PTH secretion [31,32]. The Multiple Outcomes of Raloxifene Evaluation (MORE) study involved 7,705 postmenopausal women with osteoporosis who were randomly given either raloxifene or a placebo over three years [32]. The participants were categorized into three kidney function groups based on creatinine clearance (CrCl) [32]. The study found that raloxifene significantly increased BMD at the femoral neck, especially in those with lower baseline CrCl and at the spine, irrespective of kidney function [32].

Additionally, raloxifene reduced the risk of vertebral fractures compared to placebo, though it had no significant effect on nonvertebral fractures [32]. Safety profiles were similar between groups across all kidney function categories [32]. The study concluded that raloxifene effectively increases BMD and reduces vertebral fracture risk, particularly benefiting women with mild-to-moderate CKD [32].

Anabolic Agents

Drugs that stimulate bone growth are referred to as osteoanabolic agents. Teriparatide and abaloparatide are the two osteoanabolic drugs used to treat osteoporosis. They are recombinant PTH forms that mimic PTH activity on osteoblasts [33]. However, their administration is contraindicated in CKD patients with high-turnover bone disease caused by raised PTH levels [34]. High PTH levels can lead to CKD-associated osteoporosis via increased cortical porosity and thinning due to endocortical trabecularization [35]. The long-term adverse effects of osteoanabolic drugs in CKD patients have not been investigated [36]. Because hyperparathyroidism is linked to cardiovascular calcification and mortality, osteoanabolic medications may have the same effect [37].

Teriparatide

Teriparatide is a peptide derived from PTH's first 34 amino acids [36,37]. These osteoanabolic drugs were the first approved by the FDA to prevent fractures in aged patients and patients with osteoporosis caused by glucocorticoids [32-38]. Comparing postmenopausal women treated with teriparatide for 19 months to placebo-treated postmenopausal women, biopsies of the iliac crest show a substantial increase in bone volume and thickness of the cortical bone [39]. Postmenopausal women with osteoporosis and an eGFR of 30-80 mL/minute per 1.73 m² improved their lumbar spine and femoral neck BMD in a post hoc analysis of the Fracture Prevention Trial [40]. Vertebral and nonvertebral fractures were less common in women with an eGFR of less than 80 mL/minute per 1.73 m² [39,40]. Teriparatide's pharmacokinetic safety profile in renal failure has now been investigated, demonstrating that once-weekly injections of teriparatide pose no risk of accumulation [41]. The use of teriparatide in people with intermediate-to-severe CKD and MBD has been studied in small observational studies [41,42]. In CKD stage 5D patients with biopsy-proven adynamic bone disease, teriparatide administered daily for six months increased BMD at the lumbar spine, showing significant monthly gains in both lumbar and femoral neck BMD [43]. Teriparatide given once a week to CKD stage 5D patients with hypoparathyroidism and osteoporosis improved lumbar spine BMD [44,45]. The study by Nishikawa et al. involved 33 Japanese patients with severe CKD (30 with stage 4 and three with stage 5) and osteoporosis at high risk of fracture [46]. These patients received daily subcutaneous injections of teriparatide (20 μg) for up to 24 months [46]. The objective was to assess the safety and effectiveness of teriparatide in this specific population [46]. The study found that teriparatide effectively increased BMD and bone formation markers, such as procollagen type 1 N-terminal propeptide (P1NP) [46]. No severe adverse drug reactions (ADRs) were observed, though four mild ADRs (e.g., nausea and dizziness) were reported [46]. Fractures were noted in one patient with stage 5 CKD but none in those with stage 4 CKD [46]. The study concluded that teriparatide is safe and effective in increasing BMD and bone formation in elderly female patients with severe CKD, with no new safety concerns observed [46].

Abaloparatide

Abaloparatide is a PTH-related peptide analog whose amino acids are shared by the PTH-related protein (PTHrp) [47]. Abaloparatide significantly improved BMD at the hip and femoral and lumbar spine in postmenopausal women [47-49]. Postmenopausal women on abaloparatide injection for 18 months had a 0.14 (p = 0.001) relative risk of vertebral fractures versus women receiving a placebo in the Abaloparatide Comparator Trial In Vertebral Endpoints (ACTIVE) [50]. There were no symptoms of increased osteoid, marrow fibrosis, or mineralization difficulties in postmenopausal women who were given abaloparatide for 12-18 months [50]. Moreover, compared to teriparatide, individuals taking abaloparatide had a lower deteriorated surface on histomorphometry but equivalent increases in cortical permeability [50]. These results are congruent with the findings of research trials on bone turnover markers, which reveal that the rise in serum carboxy-terminal collagen crosslinks (CTX), a bone resorption marker, was considerably less significant with abaloparatide than with teriparatide [50]. Abaloparatide reduces the chance of hypercalcemia by up to 50% compared to teriparatide [51]. No research has been published on abaloparatide in kidney failure individuals [50-52]. In people with normal renal function, its use is linked with a lower chance of elevated calcium and uric acid, making it an appealing remedy for treating patients with CKD [50-52].

Zoledronic Acid and Ibandronate

Zoledronic acid and ibandronate are other bisphosphonates used in osteoporosis management [53]. The Health Outcomes and Reduced Incidence with Zoledronic Acid Once Yearly (HORIZON) trials revealed that zoledronic acid might pose a risk of kidney failure, particularly in patients with moderate-to-severe kidney impairment [54]. However, kidney function generally returned to baseline before subsequent infusions [55,56]. Zoledronic acid and ibandronate are alternative bisphosphonates used in osteoporosis management [53]. However, the HORIZON trials raised concerns about zoledronic acid's potential nephrotoxicity, particularly in patients with moderate-to-severe kidney impairment [54]. While kidney function often returns to baseline before subsequent infusions [55,56], this finding may not fully capture the drug's long-term renal risks. Notably, studies suggest that repeated exposure to zoledronic acid can lead to cumulative kidney damage and an increased risk of chronic kidney disease (CKD) [57,58]. Therefore, caution is warranted when prescribing zoledronic acid to patients with pre-existing kidney disease or those at risk for kidney impairment. Close monitoring of renal function, including serum creatinine and estimated glomerular filtration rate (eGFR), is essential before and after each infusion [59]. Clinicians should weigh the benefits of zoledronic acid against its potential kidney risks and consider alternative treatments for patients with compromised renal function [57,58]. Zoledronic acid should not be used in patients with low creatinine clearance of below 35 mL/minute due to the risk of kidney damage [57]. Similarly, IV ibandronate has shown a safety profile similar to alendronate, with no significant difference in eGFR changes from baseline among patients with different degrees of kidney function [58].

Development of New Agents

Recent research has explored various new anti-fracture agents, revealing their potential to enhance bone mass and significantly reduce fracture risk.

Sclerostin Inhibitors

Sclerostin, a protein osteocytes produce, inhibits osteoblast activity [59]. Romosozumab, a monoclonal antibody targeting sclerostin, has demonstrated promising results in promoting bone formation [60]. The safety of romosozumab in CKD patients warrants consideration, given its potential cardiovascular risks. Studies have shown that romosozumab may increase the risk of cardiovascular events, including myocardial infarction, stroke, and cardiovascular death [61,62]. A meta-analysis of six trials found a significant 19% increase in major adverse cardiovascular events (MACE) with romosozumab compared to placebo [63]. CKD patients, already at elevated cardiovascular risk due to chronic inflammation, oxidative stress, and vascular calcification, may be particularly vulnerable to romosozumab's cardiovascular effects [64]. The FDA's black box warning for romosozumab highlights its potential cardiovascular risks [65]. Clinicians should exercise caution when prescribing romosozumab to CKD patients, carefully weighing its benefits against cardiovascular risks and closely monitoring patients for signs of cardiovascular adverse events [65,66].

Ogata et al. conducted a study showing the effectiveness and safety of romosozumab over 12 months involving 419 postmenopausal women aged 55-85 with low BMD, defined by T-scores of -2.0 or lower at the lumbar spine, femoral neck, or total hip and -3.5 or higher at each site [61]. This phase 2 multicenter, international, randomized, placebo-controlled study involved eight treatment groups [61]. The participants in the study were administered romosozumab subcutaneous injections at doses of 70 mg, 140 mg, or 210 mg, either monthly or every three months. Additionally, the participants received daily subcutaneous injections of teriparatide, a subcutaneous placebo, or an open-label active comparator, oral alendronate [61]. The primary measure assessed was the percentage difference in BMD at the lumbar spine following 12 months [61]. The results showed that all doses of romosozumab significantly increased lumbar spine BMD, with the 210 mg monthly dose leading to an 11.3% increase, compared to 0.1% with placebo, 4.1% with alendronate, and 7.1% with teriparatide [61]. Moreover, romosozumab led to notable enhancements in BMD at the femoral neck and total hip, along with transient elevations in bone formation markers and sustained reductions in a bone resorption marker [62]. A 2024 study by Adami et al. confirmed that romosozumab significantly elevated BMD and declined fracture risk in postmenopausal women with osteoporosis [63]. The study highlighted that romosozumab promotes bone formation and decreases bone resorption, making it a dual-action agent [63].

Cathepsin K Inhibitors

Odanacatib (ODN), a specific inhibitor targeting cathepsin K, has demonstrated the ability to enhance BMD and lower markers of bone resorption in postmenopausal women with low BMD during a two-year treatment period [64-68]. The LOFT study by McClung et al. included 16,713 postmenopausal women aged 45-85 with osteoporosis (BMD T-scores of -2.0 or lower but not less than -3.5) [64]. The participants were randomly assigned to one of five treatment groups, receiving either 3 mg, 10 mg, 25 mg, or 50 mg of ODN weekly or a placebo [64]. The study spanned a three-year treatment phase and a one-year extension phase [64]. During the three-year phase, the participants on the 50 mg dose of ODN saw significant BMD increases at the spine (7.9%) and total hip (7.9%), with smaller gains from baseline at these sites [64]. However, upon the discontinuation of ODN, bone loss occurred at all sites, though BMD levels remained at or above baseline [64]. Bone turnover markers showed that urinary N-telopeptide of type 1 collagen (NTx) remained suppressed by 50.5%, while bone-specific alkaline phosphatase (BSAP) remained unchanged [64]. After stopping treatment, bone turnover markers briefly spiked above baseline but returned to near-normal levels by 36 months [64]. ODN also significantly reduced the risk of new vertebral fractures, and adverse events were similar across treatment groups, with no new safety concerns during the extension phase [64]. The study concluded that three years of ODN treatment resulted in continuous BMD gains and was generally well-tolerated, with reversible effects upon discontinuation [64-69]. ODN showed promise in treating osteoporosis, but its development was halted in 2016 due to cardiovascular safety concerns [62,63]. Phase 3 trials revealed an increased risk of stroke, particularly ischemic stroke, associated with odanacatib [61,62]. The LOFT trial demonstrated a 19% increased risk of stroke (HR, 1.19; 95% CI, 1.01-1.40) and a 21% increased risk of ischemic stroke (HR, 1.21; 95% CI, 1.01-1.45) [63]. Although odanacatib significantly reduced vertebral and nonvertebral fractures, its cardiovascular risks outweighed its benefits, leading Merck to discontinue its development [64]. The FDA and European Medicines Agency (EMA) also issued warnings regarding ODN cardiovascular safety concerns [65,66]. Table 2 summarizes the characteristic features of different recent studies related to the management of osteoporosis in CKD patients.

**Table 2:** Characteristic Features of Recent Studies Related to the Management of Osteoporosis in Chronic Kidney Disease (CKD) Patients BMD: bone mineral density

Author	Year	Source of Data	Population	Sample Size, n	Results
Leng et al. [29]	2023	Meta-analysis	10,214 (all cases)	10,214	Significant reduction in fracture risk with various medications, including bisphosphonates and denosumab
Guelman et al. [67]	2023	Prospective study	264 (all cases)	264	Teriparatide significantly increased lumbar spine BMD by 13% and femoral neck BMD by 7.9% over 24 months
Tiong et al. [68]	2023	Randomized trial	278 (all cases)	278	Lanthanum carbonate significantly reduced serum calciprotein particles in patients with stage 3-4 CKD
Miller et al. [20]	2022	Post hoc analysis	Not specified	Not specified	Denosumab significantly improved BMD and reduced fracture risk compared to placebo
Hara et al. [52]	2022	Systematic review and meta-analysis	Not specified	Not specified	Bisphosphonates and denosumab were effective in reducing fracture risk and improving BMD
Toussaint et al. [66]	2020	Randomized trial	278 (all cases)	278	Sevelamer significantly reduced serum phosphate and improved vascular calcification compared to placebo

## Conclusions

Managing osteoporosis in patients with CKD requires tailored approaches due to the complex interplay between bone metabolism and renal function. Clinicians should develop personalized treatment strategies based on the CKD stage, BMD, and overall health status, with regular renal function and bone health monitoring. While DEXA scans are commonly used, advanced imaging techniques such as QCT and bone biopsies can provide more accurate assessments. The use of antiresorptive medications such as bisphosphonates and denosumab should be carefully evaluated, with close monitoring for potential nephrotoxicity and hypocalcemia. Emerging therapies such as romosozumab and cathepsin K inhibitors show promise but come with cardiovascular risks, necessitating careful consideration. Encouraging lifestyle changes that support bone health, including adequate calcium and vitamin D intake, regular exercise, smoking cessation, and fall prevention, is essential.

Further research is needed to understand osteoporosis treatments' long-term safety and efficacy in CKD patients, particularly for newer agents such as abaloparatide and romosozumab. Studies should focus on the relationship between bone quality and fracture risk in CKD patients, as traditional BMD measurements may not fully capture fracture risk. Developing CKD-specific guidelines for osteoporosis management and prioritizing patient-centered outcomes, including quality of life and functional status, are crucial. By addressing these gaps and implementing tailored management strategies, clinicians can improve outcomes for CKD patients with osteoporosis, reducing fracture risk and enhancing overall quality of life.
